# Pro‐Inflammatory c‐Met^+^ CD4 T Cells in Multiple Sclerosis

**DOI:** 10.1002/ana.78035

**Published:** 2025-09-26

**Authors:** Gautier Breville, Mahdia Benkhoucha, Ayman Rezk, Ngoc Lan Tran, Isis Senoner, Amit Bar‐Or, Patrice H. Lalive

**Affiliations:** ^1^ Department of Clinical Neurosciences, Division of Neurology University Hospital of Geneva Geneva Switzerland; ^2^ Department of Pathology and Immunology Faculty of Medicine, University of Geneva Geneva Switzerland; ^3^ Geneva Centre for Inflammation Research Faculty of Medicine, University of Geneva Geneva Switzerland; ^4^ Center for Neuroinflammation and Experimental Therapeutics, Department of Neurology Perelman School of Medicine, University of Pennsylvania Philadelphia PA USA

## Abstract

**Objective:**

Hepatocyte growth factor (HGF) binds exclusively the c‐Met surface receptor, and the HGF/c‐Met axis regulates T cell function in autoimmune diseases. We analyzed c‐Met expression on human CD4 T cells in the blood and cerebrospinal fluid (CSF) from patients with multiple sclerosis (MS) versus non‐inflammatory neurological disease (NIND), to better understand the role of CD4 T cells in MS.

**Methods:**

We recruited 34 untreated MS patients (age 28–44 years) and 13 NIND (age 34–51 years) who underwent paired blood and CSF sampling at the time of diagnosis work‐up. Phenotypic and functional CD4 T cells characterization was determined by flow cytometry and bulk RNA sequencing. Adhesion and transmigration capacities were studied to further characterize the function of c‐Met^+^ CD4 T cells.

**Results:**

c‐Met^+^ memory CD4 T cells were detected at higher levels in both blood (median of 1.98%) and CSF (5.88%) in MS compared to NIND (0.37% and 0.68%, respectively) (*p* < 0.0001). Ex vivo c‐Met^+^ CD4 T cells exhibited higher levels of GM‐CSF, interleukin (IL)‐17, interferon (IFN)‐γ, and double positive IL‐17^+^IFN‐γ ^+^ expression, compared with c‐Met^−^ CD4 T cells. c‐Met^+^ CD4 T cells expressed increased levels of integrins—Itgα4β1 (VLA‐4) and ItgαLβ2 (LFA‐1)—compared with c‐Met^−^ CD4 T cells. Anti‐Itgα4 (natalizumab) and anti‐ItgαLβ2 (odulimomab) inhibited CD4 T cell transmigration with predominant inhibition of CD4 T cells expressing c‐Met.

**Interpretation:**

These results emphasize c‐Met as an immune marker of highly pro‐inflammatory and migratory CD4 T lymphocytes in both the periphery and central nervous system of MS patients. ANN NEUROL 2026;99:261–273

Multiple sclerosis (MS) is a chronic inflammatory and neurodegenerative condition of the central nervous system (CNS), culminating in demyelination, axonal damage, and neuronal loss.^1^ An imbalance between the proportions of effector CD4 (such as Th1, Th17, and Th17.1) and regulatory CD4 T cells is reported in the peripheral blood and cerebrospinal fluid (CSF) of MS patients.^2^ These subsets also exhibit abnormalities in their functional profiles.^2^ For example, disease‐implicated pro‐inflammatory CD4 T cells express several adhesion molecules (including integrin α4) involved in migration across the blood–brain barrier (BBB),^2^, ^3^ and chemokine receptors such as CXCR3 and CCR6, which can mediate chemotaxis toward the inflamed CNS. Phenotypic and functional heterogeneity exists among disease‐implicated CD4 T cells, as revealed in high‐dimensional cytometric and scRNA‐sequencing (RNA‐seq) analyses.^2^, ^4^, ^5^, ^6^, ^7^

Our laboratory recently identified c‐Met, a tyrosine kinase receptor for hepatocyte growth factor (HGF),^8^, ^9^, ^10^, ^11^, ^12^, ^13^ as a T cell receptor (TCR)‐inducible cell surface receptor that is preferentially expressed on activated human CD4 Th17 and Th17.1 T cells.^14^ Notably, we found that in vitro polyclonal stimulation of CD4 T cells from healthy individuals results in upregulation of c‐Met expression, and c‐Met^+^ CD4 T cells had Th17 and Th17.1 phenotypes as well as an elevated expression of integrin α4 (Itgα4) compared with c‐Met^−^ CD4 T cells.^14^, ^15^ A similar functional phenotype of c‐Met^+^ CD4 T cells was apparent at the peak of experimental autoimmune encephalitis (EAE) and adoptive transfer of c‐Met^+^ CD4 T cells to naive animals resulted in more severe EAE score.^15^ Whether c‐Met^+^ CD4 T cells similarly enrich for pro‐inflammatory cells in MS patients remains unknown.

To this end, we set out to examine the phenotype and function of c‐Met^+^ CD4 T cells in paired blood and CSF samples from a cohort of untreated MS patients and sex‐ and age‐matched non‐inflammatory neurological disease (NIND) patients by using multiparametric flow cytometry, bulk RNA‐seq, and in vitro migration assays. We find that c‐Met^+^ CD4 T cells are more abundant in the blood of MS patients where they display an enhanced pro‐inflammatory phenotype and migratory potential. The latter is borne out in migration assays and more importantly by the demonstration of increased abundance of c‐Met^+^ CD4 T cells in CSF of MS patients. Our study highlights c‐Met as a marker of a pro‐inflammatory and pro‐migratory memory CD4 T cell population that is abnormal in the blood and CSF of MS patients.

## Results

### Increased Frequency of c‐Met^+^ Memory CD4 T Cells in the Blood of MS Patients

**FIGURE 1:**
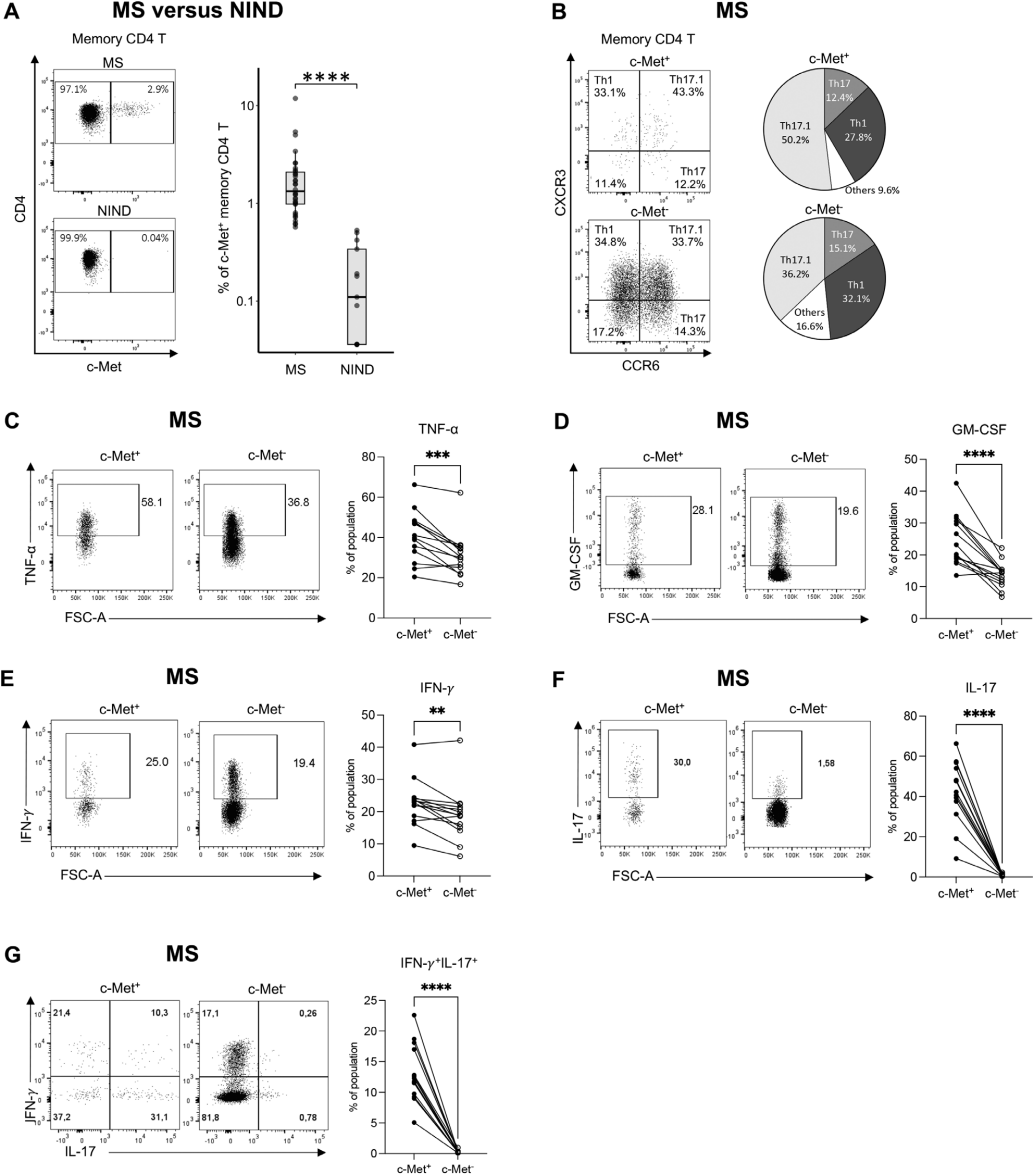
**Increased frequency of blood c‐Met** ^+^ **CD4 T cells displaying enhanced inflammatory propensity in multiple sclerosis (MS) patients**. (A) Representative flow cytometry plots of c‐Met expression on memory CD4 T cells— following exclusion of naive CD4 T cells defined as CCR7^+^CD45RA^+^—in peripheral blood of patients with MS (n = 34) and non‐inflammatory neurological disease (NIND) (n = 13). Summary graph shows elevated c‐Met expression on memory CD4 T cells in the blood of MS patients compared with NIND patients (median of 1.98% vs 0.37%; *p* < 0.0001). The memory CD4 T cells encompass central memory T cells (T_CM_, CCR7^+^CD45RA^−^), effector memory T cells (T_EM_, CCR7^−^CD45RA^−^), and terminal effector (T_EMRA_, CCR7^−^CD45RA^+^). For the box plots, the central lines indicate the median values, the top and bottom lines indicate the 75th and 25th percentiles and the whiskers represent 1.5× the interquartile range (IQR). Statistical significance was assessed using the nonparametric Wilcoxon test. (B) Representative flow cytometry plots showing the expression of CXCR3 and CCR6 on c‐Met^+^ and c‐Met^−^ CD4 T cells in the blood of MS patients (n = 34), defining CXCR3^+^CCR6^−^ (Th1), CXCR3^−^CCR6^+^ (Th17), and CXCR3^+^CCR6^+^ (Th17.1). Pie charts depict the median proportions of T cell subpopulations (Th1, Th17, and Th17.1) within c‐Met^+^ and c‐Met^−^ populations. Others refer to CXCR3^−^CCR6^−^ cells. (C–F) Representative flow cytometry plots and summary graphs showing the expression of tumor necrosis factor (TNF)‐α (C), GM‐CSF (D), interferon (IFN)‐γ (E), and interleukin (IL)‐17 (F) measured in c‐Met^+^ and c‐Met^−^ CD4 T cells in the blood of MS patients (n = 15) following short‐term stimulation (phorbol 12‐myristate 13‐acetate [PMA], ionomycin, monensin). (G) Representative flow cytometry plots and summary graph showing the co‐expression of IL‐17 and IFN‐γ in c‐Met^+^ and c‐Met^−^ CD4 T cells in the blood of MS patients (n = 15) defining IL‐17^+^ IFN‐γ ^+^ cells. *p*‐value was calculated using a paired *t* test.

We first examined the expression of c‐Met on CD4 T cells in fresh peripheral blood mononuclear cells (PBMC) obtained from untreated MS patients at the time of diagnosis and sex‐ and age‐matched NIND patients (Table 1). Flow cytometric analysis showed elevated c‐Met expression on memory CD4 T cells—defined after exclusion of naive CD4 T cells that are CCR7^+^CD45RA^+^ (flow cytometry gating strategy shown in Fig S1)—in the blood of MS patients compared with NIND patients (median of 1.98% vs 0.37%; *p* < 0.0001) (Fig 1A). The c‐Met^+^ CD4 T cell population was skewed toward Th17.1 (CXCR3^+^CCR6^+^) phenotype compared with c‐Met^−^ CD4 T cells (Fig 1B). We next probed the cytokine profile of c‐Met^+^ CD4 T cells following short‐term stimulation with phorbol 12‐myristate 13‐acetate (PMA) and ionomycin. c‐Met^+^ CD4 T cells expressed higher levels of tumor necrosis factor (TNF)‐α, GM‐CSF, interferon (IFN)‐γ, and markedly of interleukin (IL)‐17A, compared with c‐Met^−^ CD4 T cells (Fig 1C–F). The IL‐17^+^ CD4 T cells co‐expressing IFN‐γ (Th17.1) were significantly enriched among c‐Met^+^ CD4 T cells (Fig 1G). Overall, our data demonstrate that c‐Met expression defines a population of memory CD4 T cells with high pro‐inflammatory propensity that is significantly increased in the blood of untreated MS patients. In humans, such a population (IL‐17^+^ IFN‐γ ^+^) displayed higher expression of pro‐inflammatory molecules, including the gene signature reported to define murine pathogenic Th17 cells in EAE, which can more efficiently cross the BBB.^16^, ^17^, ^18^, ^19^

**TABLE 1:** Demographic and Clinical Data of Patients with Relapsing–Remitting MS and NIND

	MS (n = 34)	NIND (n = 13)	*p*
Demographic data			
Age, median (IQR), yr	35.5 (28.2–43.5)	42.0 (34.0–51.0)	0.295
F to M ratio (% of F)	21:13 (61.8)	9:4 (69.2)	0.743^*^
Clinical data			
Relapsing remitting MS, n (%)	34 (100)	–	–
Disease duration (days)	24.0 (7.0–53.0)	58.0 (13.0–238.0)	0.361
Glucocorticosteroid (%)	0 (0)	–	–
Disease modifying treatment (%)	0 (0)	–	–
CSF			
WBC/mm^3^ (IQR)	4.0 (3.0–7.0)	1.0 (0.5–1.0)	0.006
% of lymphocytes (IQR)	94.5 (93.0–96.0)	89.5 (86.5–95.8)	0.110
% of plasmocytes (IQR)	1.0 (0.0–2.0)	0.0 (0.0–0.0)	<0.001
Proteins, g/L (IQR)	0.3 (0.3–0.5)	0.3 (0.3–0.5)	0.428
IgG index (IQR)	0.9 (0.6–1.2)	0.5 (0.5–0.5)	<0.001
Albumin quotient (IQR)	4.7 (3.4–6.2)	5.6 (3.8–6.5)	0.594
Intrathecal IgG‐synthesis, n (%)	33 (97.1)	0 (0.0)	<0.001

IgG index (n < 0.7); albumin quotient (n < 8.0). *p*‐Values were calculated by Wilcoxon test or chi‐square test (*).

CSF = cerebrospinal fluid; F = female; IgG = immunoglobulin G; IQR = interquartile range; M = male; MS = multiple sclerosis; n = number; NIND = non‐inflammatory neurological diseases; yr = years.

### Transcriptomic Analysis of c‐Met^+^ and c‐Met^−^ CD4 T Cells in the Blood of MS Patients

**FIGURE 2:**
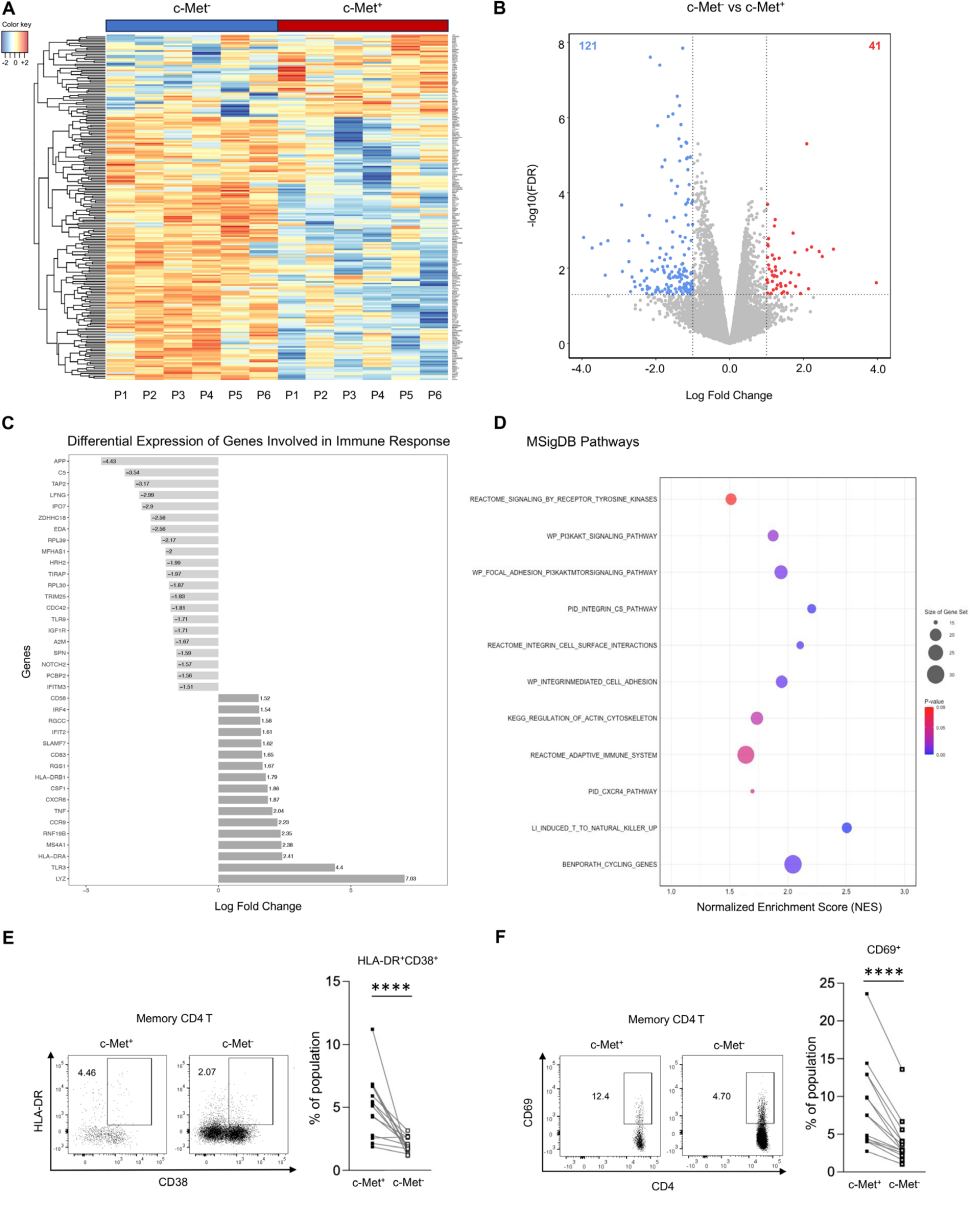
**Bulk RNA‐sequencing analysis of c‐Met** ^+^ **and c‐Met** ^**−**^ **CD4 T cells isolated from the blood of multiple sclerosis (MS) patients**. (A) Heatmap displaying 162 differentially expressed genes between c‐Met^+^ and c‐Met^−^ CD4 T cells from six MS patients. The cut‐offs were set as fold‐change >2 and *p*‐value <0.05. (B) Volcano plot showing 41 upregulated (red) and 121 downregulated (blue) genes (with false discovery rate [FDR]‐adjusted *p*‐value <0.05 and fold change >1.5) in circulating c‐Met^+^ versus c‐Met^−^ CD4 T cells isolated from the same six MS patients. (C) Differential expression of selected genes associated with immune response (Molecular Signatures Database [MSigDB]) in c‐Met^+^ and c‐Met^−^ CD4 T cells. The cut‐offs were set as fold‐change >1.5 and *p*‐value <0.05. (D) Pathway enrichment analysis in c‐Met^+^ CD4 T cells using the MSigDB and shown as normalized enrichment score (NES). Statistical comparisons were made using 2‐tailed Fisher's exact test and Benjamini‐Hochberg adjustment of *p*‐values. (E) Representative flow cytometry plots of co‐expression of HLA‐DR and CD38 on c‐Met^+^ and c‐Met^−^ CD4 T cells defining recently activated cells as HLA‐DR^+^CD38^+^ and summarized in the dot plots. n = 15 MS patients. *p*‐value was calculated using a paired *t* test. (F) Representative flow cytometry plots of CD69 expression on c‐Met^+^ and c‐Met^−^ CD4 T cells defining recently activated cells as CD69^+^ and summarized in the dot plots. n = 15 MS patients. *p*‐value was calculated using a paired *t* test. [Color figure can be viewed at www.annalsofneurology.org]

We next defined the transcriptomic signature of c‐Met^+^ CD4 T cells by bulk RNA‐seq. Given the paucity of c‐Met^+^ CD4 T cells in NIND patients, we opted to focus the transcriptomic analysis on cells obtained from untreated MS patients. Paired unstimulated c‐Met^+^ and c‐Met^−^ CD4 T cell populations were FACS‐sorted from PBMC with purities >95% (as shown in Fig S2). Our analysis identified 162 differentially expressed genes with 41 upregulated and 121 downregulated genes in c‐Met^+^ versus c‐Met^−^ CD4 T cells of untreated MS patients (Fig 2A, B). We found genes upregulated in c‐Met^+^ CD4 T cells to be associated with immune modulation (*CD58*, *IRF4*, *SLAMF7*, *CD83*, *HLA‐DRB1*, *HLA‐DRA*, *TNF*, and *TLR3*), chemotaxis (*CXCR6* and *CCR9*), and cell‐cycle regulation (*RGCC*) (see Fig 2C). The expression of *HLA‐DRB1*, *HLA‐DRA*, *CD83*, *ERG1*, *ERG2*, and *RGCC* is indicative of a higher activation and proliferative status of c‐Met^+^ CD4 T cells. This was further confirmed by flow cytometric analysis revealing an increased co‐expression of HLA‐DR and CD38 as well as CD69 on c‐Met^+^ CD4 T cells compared with c‐Met^−^ CD4 T cells, and by Gene Set Enrichment Analysis (GSEA) where there was an enrichment for cell cycling pathway (normalized enrichment score (NES): 1.97; *p* = 0.01) among c‐Met^+^ CD4 T cells (see Fig 2D–F). Interestingly, we also found an enrichment for pathways involved in cell adhesion and transmigration among c‐Met^+^ CD4 T cells including PID_Integrin_CS (NES: 2.2; *p* = 0.01), Reactome_Integrin_Cell_Surface_interactions (NES: 2.1; *p* = 0.01), and WP_IntegrinMediated_Cell_Adhesion (NES: 2.0; *p* = 0.01). Altogether, our transcriptomic analysis reveals that c‐Met^+^ CD4 T cells define an activated/proliferative population with enriched signatures for cell adhesion and transmigration.

### Migratory Potential of c‐Met^+^ CD4 T Cells in MS Patients

**FIGURE 3:**
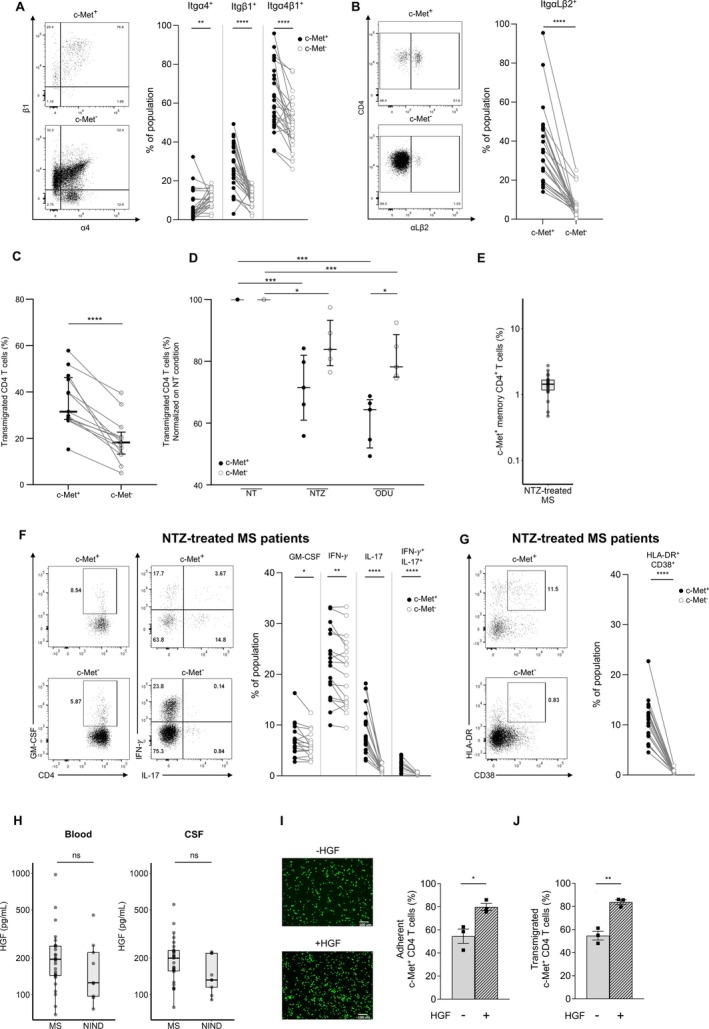
**c‐Met** ^+^ **CD4 T cells show increased expression of VLA‐4 and LFA‐1, and are associated with enhanced migratory capacities in the blood of multiple sclerosis (MS) patients**. Expression of Itgα4, Itgβ1, and Itgα4β1 (VLA‐4) (A) and ItgαLβ2 (LFA‐1) (B) was measured on c‐Met^+^ and c‐Met^−^ memory CD4 T cells as shown in representative flow cytometry plots and summarized in dot plots (n = 25 MS patients). (C) Equal numbers of highly purified c‐Met^+^ and c‐Met^−^ CD4 T cells were cultured on a monolayer of tumor necrosis factor alpha (TNF‐α)‐activated human umbilical vein endothelial cells (HUVEC) seeded in the upper chamber with CXCL12 added to the bottom chamber. Migrated c‐Met^+^ and c‐Met^−^ CD4 T cells harvested in the bottom chamber were quantified by flow cytometry after 4 hours (n = 13 MS patients). (D) CD4 T cell populations were pre‐treated with natalizumab (NTZ) (100μg/ml) or odulimomab (ODU) (100μg/ml) or matching isotype control before assessing migration across a monolayer of TNF‐α activated HUVEC cells. Harvested CD4 T cells found in the bottom chamber were quantified by flow cytometry. n = 5 MS patients. Normalized data are shown, with central lines indicating median values after data normalization to the untreated condition, and with top and bottom lines indicating the 75th and 25th percentiles, respectively. Statistical significance was assessed using the nonparametric Wilcoxon test. (E) Frequency of c‐Met^+^ memory CD4 T cells in peripheral blood of NTZ‐treated MS patients (n = 20); data shown as median with interquartile range (IQR). (F) Representative flow cytometry plots and summary graph of cytokines production by c‐Met^+^ and c‐Met^−^ CD4 T cells in the blood of NTZ‐treated individuals (n = 20); *p < 0.05, **p < 0.01, ****p < 0.0001, paired *t* test. (G) Representative flow cytometry plots and summary graph of activation markers expression on c‐Met^+^ and c‐Met^−^ CD4 T cells in blood of NTZ‐treated individuals (n = 20); ****p < 0.0001, paired *t* test. (H) Hepatocyte growth factor (HGF) concentrations in blood and cerebrospinal fluid (CSF) of MS patients (n = 34) and non‐inflammatory neurological disease (NIND) controls (n = 11); p = ns (nonparametric unpaired Wilcoxon test). (I) Representative fluorescence images (left) and quantification (right) of c‐Met^+^ CD4 T cell adhesion assay in the presence or absence of pre‐treatment with HGF; *p < 0.05, nonparametric unpaired Wilcoxon test. (J) Quantification transmigrated c‐Met^+^ CD4 T cells with (+) or without (−) pretreatment with HGF, showing enhanced migration and adhesion in the presence of HGF (n = 3); **p < 0.01, nonparametric unpaired Wilcoxon test. [Color figure can be viewed at www.annalsofneurology.org]

We previously showed that in vitro‐induced c‐Met^+^ CD4 T cells upregulated Itgα4,^14^, ^15^ and our RNA‐seq analysis pointed to an enrichment of pathways associated with cell migration and adhesion in c‐Met^+^ CD4 T cells. Flow cytometric analysis revealed higher expression of Itgα4β1 (VLA‐4) and ItgαLβ2 (LFA‐1) on c‐Met^+^ CD4 T cells compared with c‐Met^−^ CD4 T cells in MS patients (Fig 3A, B), 2 molecules involved in the migration of T cells across endothelial cells.^20^ To validate the functional relevance of differential integrin expression, highly purified c‐Met^+^ and c‐Met^−^ CD4 T cells were cultured onto a monolayer of TNF‐α‐activated human umbilical vein endothelial cells (HUVEC) in the presence of CXCL12 in the bottom chamber. CXCR4, receptor for CXCL12, expression was similar between c‐Met^+^ and c‐Met^−^ CD4^+^ T cells (Fig S3). We found that c‐Met^+^ CD4 T cells migrated more readily than c‐Met^−^ CD4 T cells (see Fig 3C). We next pre‐treated the different CD4 T cell populations with either natalizumab (anti‐Itgα4, NTZ) or odulimomab (anti‐LFA‐1, ODU) to examine the contributions of VLA‐4 and LFA‐1 to their migration capacity across TNF‐α‐treated HUVEC. Pre‐treatment with either monoclonal antibody resulted in reduced migration of both CD4 T cell populations across the endothelium with a more profound effect observed for c‐Met^+^ CD4 T cells than their c‐Met^−^ counterparts (see Fig 3D).

To determine the phenotypic and functional characteristics of c‐Met^+^ memory CD4 T cells in the context of NTZ therapy, we recruited 20 stable MS patients on long‐term NTZ treatment (>12 months) (Fig 3E) and compared their circulating c‐Met^+^ memory CD4 T cell frequency with those of untreated MS patients (n = 34) (see Fig 1A). Median frequency of c‐Met^+^ memory CD4 T cell in NTZ cohort was 1.48% compared to 1.98% in untreated MS patients at the time of relapse (*p* = 0.115) yet remaining higher than in NIND cohort (0.37%; *p* < 0.001). These data confirmed the sustainable blood level of c‐Met^+^ CD4 T cells under long‐term NTZ treatment. Functional profiling revealed that c‐Met^+^ CD4 T cells of NTZ‐treated patients expressed higher levels of pro‐inflammatory cytokines—GM‐CSF, IFN‐γ, and IL‐17—compared to their c‐Met^−^ counterparts and had a more activated phenotype denoted by co‐expression of HLA‐DR and CD38 (see Fig 3F, G). These findings indicate that c‐Met expression consistently defines a functionally distinct, pro‐inflammatory memory CD4 T cell population that persists in NTZ‐treated MS patients at levels comparable to those observed in MS patients during relapse.

Given that HGF is the cognate ligand for c‐Met, we next measured HGF concentrations in matched plasma and CSF samples from patients with MS and NIND controls. We found that HGF levels were comparable between groups in both plasma and CSF (see Fig 3H). To assess the functional impact of HGF–c‐Met signaling on CD4 T cell function, we conducted in vitro adhesion and transmigration assays using c‐Met^+^ CD4 T cells, which were FACS‐purified following polyclonal stimulation of PBMC with α CD3/α CD28 with or without exogenous HGF for 3 days. c‐Met^+^ CD4 T cells pre‐treated with HGF adhered more to endothelial HUVEC monolayers, as visualized by fluorescence microscopy and confirmed by quantification (see Fig 3I) (*p* < 0.05). Similarly, pre‐treated c‐Met^+^ CD4 T cells exhibited enhanced migration across HUVEC monolayers (see Fig 3J) (*p* < 0.01). Altogether, our data indicate that c‐Met^+^ CD4 T cells have an enhanced migratory potential across the inflamed endothelium that is partially dependent on VLA‐4 and LFA‐1 and is enhanced by HGF.

### Increased Frequency of c‐Met^+^ Memory CD4 T Cells in the CSF of MS Patients Correlates with Expanded Disability Status Scale Score

**FIGURE 4:**
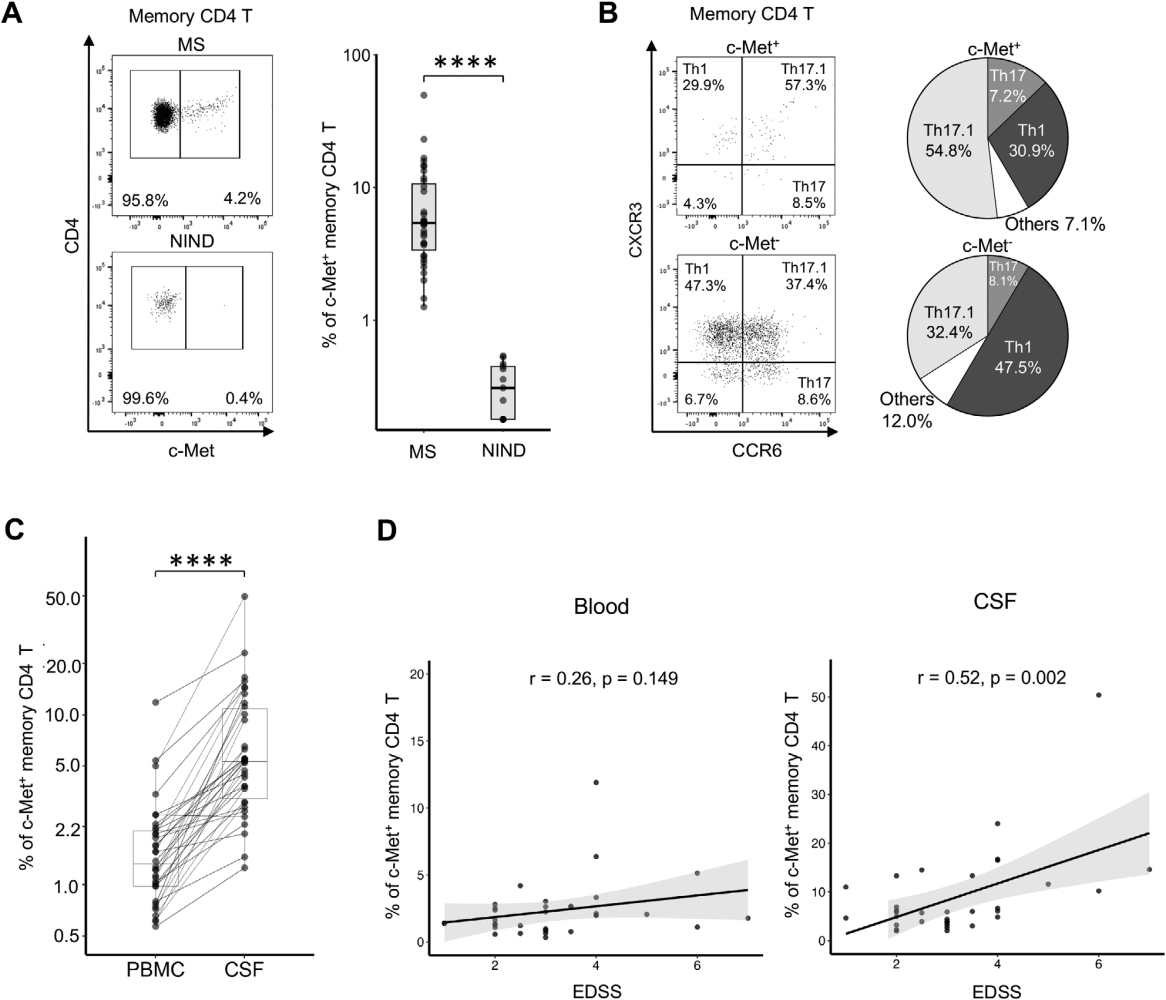
**Increased frequency of c‐Met** ^+^ **memory CD4 T cells in the cerebrospinal fluid (CSF) of multiple sclerosis (MS) patients**. (A) Representative flow cytometry plots of c‐Met expression on memory CD4 T cells in CSF from patients with MS (n = 34) and non‐inflammatory neurological disease (NIND) (n = 13). Summary graph shows elevated c‐Met expression on memory CD4 T cells in CSF of MS patients compared with NIND patients (median of 5.88% vs. 0.68%; *p* < 0.0001). The memory CD4 T cells encompass central memory T cells (T_CM_, CCR7^+^CD45RA^−^), effector memory T cells (T_EM_, CCR7^−^CD45RA^−^) and terminal effector (T_EMRA_, CCR7^−^CD45RA^+^). For the box plots, the central lines indicate the median values, the top and bottom lines indicate the 75th and 25th percentiles, and the whiskers represent 1.5× the interquartile range (IQR). Statistical significance was assessed using the nonparametric Wilcoxon test. (B) Representative flow cytometry plots showing the expression of CXCR3 and CCR6 on c‐Met^+^ and c‐Met^−^ CD4 T cells, defining CXCR3^+^CCR6^−^ (Th1), CXCR3^−^CCR6^+^ (Th17) and CXCR3^+^CCR6^+^ (Th17.1). Pie charts depict the median proportions of T cell subpopulations (Th1, Th17, and Th17.1) within c‐Met^+^ and c‐Met^−^ populations in the CSF. The remaining population (CXCR3^−^CCR6^−^) is referred to here as “Others.” n = 34 MS patients. (C) Quantification of the frequency of c‐Met^+^ memory CD4 T cells found in paired peripheral blood mononuclear cells (PBMC) and CSF samples from MS patients (n = 34). For the box plots, the central lines indicate the median values, the top and bottom lines indicate the 75th and 25th percentiles, respectively, the whiskers represent 1.5× the interquartile range (IQR). Statistical significance was assessed using the nonparametric Wilcoxon test. Flow cytometric analysis showed more significantly elevated c‐Met expression on memory CD4 T cells in the CSF of MS patients compared with blood (median of 5.88% vs 1.98%; *p* < 0.0001). (D) Frequency of c‐Met^+^ CD4 T cells in PBMCs (left; r = 0.26, *p* = 0.149) and in CSF (right; r = 0.52, *p* = 0.002) plotted against Expanded Disability Status Scale (EDSS) scores. Each dot represents an individual MS patient. Shaded areas represent 95% confidence intervals for the regression line (Spearman correlation analysis).

Our data highlight blood c‐Met^+^ CD4 T cells as a highly pro‐inflammatory population that can more efficiently migrate across the inflamed endothelium in vitro. This prompted us to examine whether c‐Met^+^ CD4 T cells were similarly efficient at infiltrating the inflamed CNS in MS using flow cytometric analysis of CSF from MS and NIND patients. c‐Met^+^ memory CD4 T cells were increased in the CSF of MS when compared with NIND patients (median of 5.88% vs 0.68%; *p* < 0.0001) (Fig 4A, see gating strategy in Fig S4). Phenotypic characterization revealed that c‐Met^+^ CD4 T cells in the CSF predominantly exhibited a Th17.1 phenotype in MS patients (Fig 4B). Finally, analysis of paired blood and CSF samples revealed an enrichment of c‐Met^+^ CD4 T cells in the CSF in MS patients (median of 5.88% vs 1.98%, Fig 4C). c‐Met^+^ CD4 T cells were skewed toward a central memory T cell (T_CM_) phenotype, whereas c‐Met^−^ CD4 T cells were skewed toward an effector memory T cell (T_EM_) phenotype, in the blood and in the CSF (Fig S5). Finally, we assessed the relationship between Expanded Disability Status Scale (EDSS) at the time of relapse and the frequencies of c‐Met^+^ CD4 T cells in blood and CSF of untreated MS patients. Although no significant correlation was observed between the frequency of blood c‐Met^+^ CD4 T cells and EDSS (r = 0.26, *p* = 0.149), a positive correlation was detected with CSF c‐Met^+^ CD4 T cells, after adjustment for CSF white blood cell levels (r = 0.52, *p* = 0.002) (see Fig 4D). Taken together, we show that c‐Met^+^ CD4 T cells are a subset of pro‐inflammatory cells with enhanced migratory propensity toward the CNS in MS patients. Their enrichment in the CSF—particularly among patients with greater clinical disability—supports a potential contribution to CNS‐compartmentalized inflammation.

## Discussion

In this study, we functionally characterize c‐Met^+^ memory CD4 T cells in untreated MS patients and provide evidence for their enhanced pro‐inflammatory and migratory capacities. We find abnormally increased frequencies of c‐Met^+^ memory CD4 T cells in the blood and CSF of untreated MS patients and demonstrate that blood c‐Met^+^ CD4 T cells of patients produce higher levels of TNF‐α, GM‐CSF, IFN‐γ, and IL‐17. c‐Met^+^ CD4 T cells also migrate more readily across the inflamed endothelium in vitro, partially dependent on VLA‐4 and LFA‐1. Overall, we highlight c‐Met as a marker of pro‐inflammatory CD4 T cells in untreated MS patients, both in the periphery and the CNS.

Several memory CD4 T cell populations, including CXCR3^+^CCR6^+^, CD161^+^, MCAM^+^, CCR5^+^, and CXCR4^+^ cells, exhibit enriched pro‐inflammatory cytokine expression^4^, ^21^, ^22^, ^23^, ^24^ and the frequencies of these populations have been reported to be elevated in the blood and/or CSF of MS patients.^4^, ^21^, ^22^ Our data expand on the phenotypic heterogeneity of memory CD4 T cells by identifying c‐Met as a marker for sub‐populations of CXCR3 and CCR6 expressing cells in the blood and CSF of untreated MS patients. This is also reflected in profiling cytokine production, as c‐Met^+^ CD4 T cells are enriched in IFN‐γ ^+^IL‐17^+^ cells following short‐term polyclonal stimulation. Our findings are particularly relevant as CXCR3^+^CCR6^+^ and IFN‐γ ^+^IL‐17^+^ CD4 T cells—commonly referred to as Th17.1 cells—are recognized as a pathogenic Th17 subset with high pro‐inflammatory and CNS migratory potential.^17^, ^23^, ^24^, ^25^, ^26^

c‐Met^+^ memory CD4 T cells have an enhanced capacity to migrate to inflamed tissues. We find an enrichment for migration and adhesion pathways in RNA‐seq analysis, which are confirmed by flow cytometry analysis, revealing higher expression of VLA‐4 and LFA‐1. These two molecules play an important role in T cell migration across the BBB, facilitating arrest, crawling, and diapedesis.^20^ Our in vitro data support the involvement of VLA‐4 and LFA‐1 in the transmigration of c‐Met^+^ CD4 T cells across activated endothelial cells. We note, however, that the blocking effects observed with NTZ and ODU are incomplete, which hints at other adhesion molecules playing a role in this process. c‐Met^+^ CD4 T cells are additionally equipped to respond to the gradient of chemokines guiding them to the inflamed CNS via their expression of CXCR3, CCR6, and CXCR6. Previous studies have shown that CXCL10 levels are higher in the CSF/CNS of MS patients,^27^, ^28^, ^29^, ^30^ and CCL20 is expressed by epithelial cells in the choroid plexus in MS patients.^31^ The expression of CXCR6 also enriches for pathogenic Th17 cells^32^ and is found on clonally expanded CD4 T cells in MS CSF.^33^ It is therefore concordant that c‐Met^+^ memory CD4 T cells are more abundant in the CSF of untreated MS patients compared to controls, where they maintain a Th17.1 phenotype and are also relatively more enriched in the CSF compared with the blood of MS patients. Finally, the denotation of c‐Met^+^ CD4 T cells as pro‐inflammatory is also supported by enhanced EAE disease severity in mice receiving c‐Met^+^ CD4 T cells, a phenotype dependent on Itgα4.^15^

In stable MS patient treated by NTZ treatment, we observed that c‐Met^+^ memory CD4 T cell population persists in the peripheral blood. Although overall frequencies were not significantly different from those observed in untreated relapsing MS patients at the time of MS relapse, they remained markedly elevated compared to NIND controls. This corroborates that NTZ, although effective at blocking lymphocyte trafficking across the BBB, does not eliminate circulating pro‐inflammatory cells subset.^34^ Notably, c‐Met^+^ CD4 T cells from NTZ‐treated patients retained a highly inflammatory phenotype, characterized by the expression of GM‐CSF, IFN‐γ, and IL‐17, cytokines implicated in MS pathogenesis. Additionally, the elevated expression of activation markers HLA‐DR and CD38 in c‐Met^+^ compared to c‐Met^−^ CD4 T cells further supports their potential involvement in sustained immune activation. Together, these findings suggest that c‐Met expression marks a functionally distinct memory CD4 T cell population that may remain poised for pathogenic activity even under integrin‐blocking therapy. This persistent proinflammatory population may contribute to rebound inflammatory activity observed after NTZ discontinuation.^35^

Interestingly, the level of HGF—known as the cognate ligand of c‐Met—was similar in MS versus NIND patients, as previously described.^36^, ^37^ We found that HGF enhances both adhesion and trans‐endothelial migration of c‐Met^+^ CD4 T cells, supporting a role for the HGF/c‐Met axis in promoting T cell recruitment to CNS tissue. These findings are consistent with prior work in other inflammatory settings, which demonstrated that c‐Met signaling promotes T cell trafficking via autocrine chemokine release.^38^

However, the functional role of c‐Met on CD4 T cells has yet to be fully elucidated. Rather than implying that c‐Met directly regulates the inflammatory features of memory CD4 T cells in MS, our data indicate that CD4 T cells expressing c‐Met represent a more inflammatory and migratory subset within the context of MS, suggesting they may be involved in disease mediation, and hence, with the translational implication that future selective targeting of c‐Met expressing CD4 T cells may provide a safer therapeutic approach in the long‐term. Although prior work has shown that the HGF/c‐Met axis can play a role in cellular migration,^38^ of particular interest may be the work in the field of neurobiology that has established a role for the HGF/c‐Met axis in promoting cell survival.^39^, ^40^, ^41^ This principle may extend to the field of immunology, where prior work described a subset of memory B cells that expresses the nerve growth factor (NGF) receptor and receives NGF signals that promote their survival, which can be an elegant way for immune cell memory to be sustained by providing selected memory cells with a survival advantage.^42^ We speculate that expression of c‐Met on pro‐inflammatory and migratory memory CD4 T cells may contribute to their longer‐term survival and potentially more substantial contribution to the disease process, and more selective targeting the c‐Met^+^ subset of CD4 T cells may offer a novel strategy aimed at limiting long‐term persistence and possible pathogenic contribution of these cells.

Finally, our findings support a potential association between neurological disability and the intrathecal accumulation of c‐Met^+^ CD4 T cells during relapse. The absence of correlation in blood, contrasted with the significant association observed in CSF, suggesting that c‐Met^+^ CD4 T cells may preferentially contribute to CNS‐compartmentalized immune activity in more disabled patients.

In conclusion, c‐Met is a marker of pro‐inflammatory and migratory memory CD4 T cells enriched in both blood and CSF of MS patients. Future studies could assess the antigenic specificity of c‐Met^+^ CD4 T cells. Moreover, c‐Met^+^ CD4 T cells may be a particularly relevant cell subset to target therapeutically, using blockade of c‐Met receptor itself or selectively targeting c‐Met^+^ CD4 T cells for killing. Selectively targeting c‐Met^+^ CD4 T cells may be effective in MS without limiting other cell trafficking and, therefore, limiting the risk of infections such as progressive multifocal leukoencephalopathy.

## Materials and Methods

### Ethics Approval and Consent to Participate

This study was approved by the institutional review board of the Geneva University Hospitals (protocol 2020‐02724—approved November 2020). Written informed consent was obtained from all included patients and controls. This study was conducted according to the World Medical Association Declaration of Helsinki.

### Study Participants

Between November 2020 and October 2022, at the Geneva University Hospitals, we recruited 47 patients who were either diagnosed with relapsing–remitting MS (2017 Mc Donald criteria), or identified as having NIND. These patients, evaluated by a board‐certified neurologist (Neurostatus‐EDSS certified), underwent parallel lumbar punctures, and paired blood tests. Table 1 summarizes the clinical parameters and routine laboratory findings from patients diagnosed with MS (n = 34) and NIND (n = 13). Among the 13 patients with NIND, we included patients with functional neurological disorder (n = 6), idiopathic intracranial hypertension (n = 4), cryptogenic epileptic seizures (n = 2), and mild cognitive impairment (n = 1). The MS group had a median age of 35.5 years (interquartile range [IQR]: 28.3–43.5), whereas the NIND group had a median age of 42.0 years (IQR: 34.0–51.0). Age, gender distribution (61.8% females in MS vs 69.2% in NIND), the time from the clinical onset to lumbar puncture, CSF protein levels, and the CSF albumin quotient all showed no significant statistical difference between the 2 groups. As expected, the MS group exhibited a higher CSF white blood cell count, with a median of 4.0 (IQR: 3.0–7.0) compared to 1.0 (IQR: 0.5–1.0) in the NIND group (*p* = 0.006). Intrathecal immunoglobulin G synthesis was positive in 97.1% of the MS group, contrasting with none in the NIND group (*p* < 0.001).

We also prospectively recruited 20 patients with a confirmed diagnosis of relapsing–remitting MS according to the 2017 McDonald criteria, followed at the Geneva University Hospitals. All participants had been receiving NTZ therapy for more than 12 consecutive months at the time of inclusion. All these patients were stable (no clinical relapse, and no brain magnetic resonance imaging activity). These patients constituted the NTZ‐treated cohort and were included for immunophenotyping analysis of circulating c‐Met^+^ memory CD4 T cells.

### Mononuclear Cell Isolation from Whole Blood and CSF

Venous blood was collected in vacutainer K2E tubes containing EDTA (Becton Dickinson, BD Biosciences, San Jose, CA, USA) and CSF was collected in 15ml Falcon tubes via lumbar puncture from MS and NIND patients. CSF was collected and kept on ice until processing. CSF was centrifuged at 400*g* for 10 minutes. The cell pellet was gently re‐suspended in ice cold phosphate‐buffered saline (PBS) (Thermo‐Fisher 10010072) solution for the flow cytometry staining, which was performed within 2 hours after the lumbar puncture. Whole blood was processed for the isolation of PBMC by density gradient centrifugation using Ficoll Paque Plus (GE Healthcare). A fraction of PBMC was resuspended in ice cold PBS for flow cytometric analysis and the remaining cells were cryopreserved using a solution of 10% dimethyl sulfoxide in fetal bovine serum (Gibco) and stored in a liquid nitrogen tank.

### Flow Cytometry Staining

Paired PBMC and CSF samples were stained with fluorophore conjugated antibodies detailed in Table S1 for 30 minutes at room temperature. A FMO control for c‐Met staining was included. Viability was determined using 4′,6‐diamidino‐2‐phenylindole (DAPI) (Invitrogen). Data was acquired on a LSR Fortessa (BD Biosciences).

Cryopreserved cells were thawed according to standard operating procedures and were rested overnight. Next, 10^6^ cells were stimulated with phorbol myristate acetate (50ng/ml; Sigma Aldrich) and ionomycin (500ng/ml; Sigma Aldrich) in the presence of brefeldin A (10μg/ml; BD Biosciences) or left unstimulated for 4 hours. Cells were harvested, stained with a fixable aqua dead cell stain (Thermo Fisher Scientific) before staining for cell surface proteins (Table S1). Cells were fixed and permeabilized using BD Cytofix/Cytoperm (BD Biosciences). Cells were then stained for intracellular cytokines (Table S1) before data acquisition on BD LSR Fortessa (BD Biosciences). Data was analyzed using FlowJo (BD Biosciences).

### Cell Sorting of c‐Met^+^ and c‐Met^−^ CD4 T Cells

CD4 T cells were magnetically isolated from PBMC using CD4 microbeads (Miltenyi Biotec). Isolated cells were stained for anti‐human CD3 (UCHT1, Biolegend), anti‐human CD4 (SK3, BD Biosciences), anti‐human c‐Met (95,106, R&D), and DAPI before cell sorting on a FACSAria II. Sorted cells had purities >95% and were used for the transmigration assay and RNA isolation for bulk RNA‐seq (Fig. S2).

### Detection of HGF in Plasma and CSF

Soluble HGF concentrations were measured in plasma and CSF samples from patients with MS and NIND using a commercially available enzyme‐linked immunosorbent assay kit (Human HGF DuoSet ELISA, R&D Systems) according to the manufacturer's instructions. Optical density was read at 450nm using an Infinite M200 microplate reader (Tecan, Männedorf, Switzerland), and data were analyzed using Magellan software (version 7.2). Paired plasma and CSF samples were collected at the same time point. Group comparisons were performed using a nonparametric unpaired Wilcoxon test.

### CD3/CD28 and HGF Stimulation

PBMCs were isolated from healthy donors by density gradient centrifugation using Ficoll‐Paque PLUS (GE Healthcare, Life Science). Cells were resuspended in RPMI 1640 medium supplemented with 10% fetal bovine serum, 4mM L‐glutamine, 0.1% β‐mercaptoethanol, L‐asparagine, L‐arginine (Sigma‐Aldrich), 25mM Hepes buffer, 50U/ml penicillin, and 50μg/ml streptomycin (Life Technologies). Cells were activated with anti‐CD3/CD28 Dynabeads (Thermo Fisher Scientific; 1:1 bead‐to‐cell ratio) and 100IU/ml IL‐2 (Peprotech) for 72 hours at 37°C and 5% CO₂. For HGF treatment conditions, cultures were supplemented with 50ng/ml recombinant human HGF (R&D Systems) for the entire activation period. Following activation, c‐Met^+^ CD4 T cells were isolated using a FACSAria cell sorter (BD Biosciences) based on co‐staining with anti‐CD4 and anti‐c‐Met antibodies. Cell purity was consistently >99% as verified by flow cytometry. Sorted c‐Met^+^ CD4 T cells were then incubated with 5μM CFSE (Invitrogen) in PBS for 20 minutes at 37°C to enable tracking of fluorescence. Cells were subsequently washed twice with pre‐warmed PBS, counted, and assessed for fluorescence intensity by flow cytometry to confirm successful labeling. Equal numbers of CFSE‐labeled c‐Met^+^ CD4 T cells from each condition (± HGF) were used in downstream adhesion assays.

### Cell Adhesion Assay

HUVECs (Lonza) were seeded into 96‐well plates and grown to confluence in EGM‐2 medium (Lonza). Confluent monolayers were stimulated for 24 hours with 10ng/ml TNF‐α (Peprotech) to mimic an inflamed endothelium. c‐Met^+^ CD4 T cells with or without prior HGF exposure were seeded onto the HUVEC monolayers at 2 × 10^5^ cells/well and incubated for 1 hour at 37°C. Wells were gently washed 3 times with pre‐warmed PBS to remove non‐adherent cells. Five random fields per well were imaged using a ZOE Fluorescent Cell Imager (Bio‐Rad). Quantification of adherent cells was performed using CellProfiler software (Broad Institute). Data are presented as percentage of adhesive cells relative to input.

### Transmigration Assay

Transwell migration assay was performed to assess the transmigration of c‐Met^+^ and c‐Met^−^ CD4 T cells through a monolayer of HUVEC treated with TNF‐α (10ng/ml; eBioscience) in the presence of CXCL12 (100ng/ml; eBioscience) added to the bottom chamber. TNF‐α‐treated HUVEC were coated on the transwell chamber (24‐well insert, diameter: 6.5mm, pore size: 5μm; Corning, Vitaris AG). Sorted c‐Met^+^ or c‐Met^−^ CD4 T cells were seeded into the top chamber. The exact same number of c‐Met^+^ or c‐Met^−^ T cells were seed into the top chamber (ranging between 10,000 and 20,000 cells in between experiments). The plates containing the transwell chambers were then incubated for 4 hours at 37°C to allow cell attachment and transmigration. Where indicated, sorted CD4 T cell populations were incubated with either anti‐Itgα4 (10ug/ml; Biogen) or anti‐ItgαL (10μg/ml; eBioscience) for 1 hour, then washed with PBS to remove excess antibody and centrifuged at 400*g* for 10 minutes before the transmigration assay. CD4 T cells present in the lower chamber were harvested and the count of transmigrated cells was determined by flow cytometry using fluorescent counting beads (Thermo Fisher Scientific).

### RNA Isolation and Bulk‐RNA Sequencing

FACS‐sorted T cell subsets were washed with phosphate‐buffered saline (PBS) and resuspended in RNA lysis solution with 1% dithiothreitol (DTT). Total RNA was extracted using the Nucleospin RNA kit (Macherey‐Nagel). Complementary DNA (cDNA) was synthesized from total RNA using a reaction mix containing Tris‐aminoMethane (200mM), KCl (500mM), MgCl_2_ (0.2M; Sigma‐Aldrich), DTT (100mM; Invitrogen), random hexamers (50mM; Invitrogen), oligo (dT) 15 primer (100mg/ml; Promega), dNTP mix (10mM; Promega), RNAsin (40U/ml; Promega), and SuperScriptTM II (200U/ml; Invitrogen). After incubation at 42°C for 50 minutes and inactivation at 99°C for 3 minutes, cDNA was diluted and stored at −20°C until use.

On the FACS‐sorted c‐Met^−^ and c‐Met^+^ CD4 T cells, we performed a Bulk‐RNA sequencing. The SMART‐Seq v4 kit from Clontech was used for the reverse transcription and cDNA amplification according to manufacturer's specifications, starting with 1ng of total RNA as input. A total of 200pg of cDNA were used for library preparation using the Nextera XT kit from Illumina. Library molarity and quality was assessed with the Qubit and Tapestation using a DNA High sensitivity chip (Agilent Technologies). Libraries were sequenced on a NovaSeq 6,000 Illumina sequencer for SR100 reads for an average of around 35,000,000 reads/sample.

We used GSEA to identify enriched biological themes within our dataset. Initially, differentially expressed genes (DEGs) were identified based on specific criteria (eg, fold‐change and *p*‐value thresholds). DEGs were then ranked based on their expression levels. Using the ranked list, GSEA was performed to determine whether sets of genes associated with specific biological processes, molecular functions, or cellular components were statistically overrepresented. We used the Molecular Signatures Database for gene set annotations. Enrichment scores, nominal *p*‐values, and false discovery rate were calculated to assess the significance of gene set enrichment.

### Statistical Analyses

Statistical analyses were carried out using R and GraphPad Prism Software, version 9.0. We used the paired Student *t* test or Wilcoxon matched‐pairs signed rank test to compare c‐Met^+^ and c‐Met^−^ CD4 T cells of the same patient among the 2 groups (MS and NIND). Experimental data are depicted as mean (standard error of the mean) or median (IQR). *p*‐Values <0.05 were considered significant.

## Author Contributions

G.B., M.B., N.L.T., and P.H.L. contributed to the conception and design of the study; G.B., M.B., A.R., N.L.T., and I.S. contributed to the acquisition and analysis of data; G.B., M.B., A.R., N.L.T., I.S., A.B.O., and P.H.L. contributed to drafting the text or preparing the figures. All authors approved the manuscript.

## Potential Conflicts of Interest

Nothing to report.

## Supporting information


**Table S1.** Flow cytometry antibodies.

**Figure S1.** Representative gating strategy of blood c‐Met^+^ memory CD4 T cells. Freshly isolated PBMC were stained with antibodies directed against CD3, CD4, CCR7, CD45RA and c‐Met to identify c‐Met^+^ and c‐Met^−^ among memory (non‐CCR7^+^CD45RA^+^) CD4 T cells.

**Figure S2.** Representative purity of fluorescence‐activated cell sorting (FACS) of c‐Met^+^ and c‐Met^−^ CD4 T cells. Freshly isolated PBMC were stained with antibodies directed against CD3, CD4 and c‐Met to sort c‐Met^+^ and c‐Met^−^ CD4 T cells. Sorted populations were examined for their purity, which routinely exceeded >95%.

**Figure S3.** Similar CXCR4 expression on c‐Met^+^ and c‐Met^−^ CD4 T cells in the blood of MS patients. CXCR4 expression was measured on the surface of c‐Met^+^ (solid line) and c‐Met^−^ (dotted line) CD4 T cells in cryopreserved PBMC from three untreated MS patients. An isotype antibody (shaded gray) was used as a control for CXCR4 staining.

**Figure S4.** Representative gating strategy of CSF c‐Met^+^ memory CD4 T cells. Fresh CSF cells were stained with antibodies directed against CD3, CD4, CCR7, CD45RA and c‐Met to identify c‐Met^+^ and c‐Met^−^ among memory (non‐CCR7^+^CD45RA^+^) CD4 T cells.

**Figure S5.** Distribution of naive and memory subsets among c‐Met^+^ and c‐Met^−^CD4 T cells in the blood and CSF of MS patients. Representative staining of CCR7 and CD45RA on c‐Met^+^ and c‐Met^−^ CD4 T cells in PBMC (A) and CSF (B), defining CCR7^+^CD45RA^+^ (naive), CCR7^+^CD45RA^−^ (T_CM_), CCR7^−^CD45RA^−^ (T_EM_) and CCR7^−^CD45RA^−^ (T_EMRA_). (C) Relative frequency of naive and memory CD4 T cell subsets among c‐Met^+^ and c‐Met^−^ CD4 T cells in paired PBMC and CSF samples obtained from MS patients (*n* = 15).

## Data Availability

All raw data are available on reasonable request to the corresponding author G.B. The transcriptomic raw data supporting the findings of this study are publicly available in the National Center for Biotechnology Information Gene Expression Omnibus (GEO) under accession number GSE305678.
