# Supplementary material for: Pro‐Inflammatory c‐Met+ CD4 T Cells in Multiple Sclerosis
Source: Ann Neurol. 2025 Sep 26;99(1):261–73. doi: 10.1002/ana.78035 (PMC12946607; doi:10.1002/ana.78035)
Supplement: Supplementary file 1 — Table S1. Flow cytometry antibodies. [file ANA-99-261-s005.docx]

**SUPPLEMENTARY TABLE**

**Table S1: Flow cytometry antibodies**

| **Antibodies** | **Species reactivity** | **Fluorochrome** | **Clone** | **Supplier** | **RRID number** |
| --- | --- | --- | --- | --- | --- |
| CD4 | Anti-human | BUV737 | SK3 | BD Biosciencess | AB_2870080 |
| c-Met | Anti-human | FITC | 95106 | R&D | unknown |
| c-Met | Anti-humain | APC | 95106 | R&D | unknown |
| CD38 | Anti-human | BUV395 | HB7 | BD Biosciences | AB_2744372 |
| CCR7 | Anti-human | PE | 150503 | BD Biosciences | AB_2828646 |
| CD69 | Anti-human | APC-R700 | FN50 | BD Biosciences | AB_2744449 |
| HLA-DR | Anti-human | PerCPcy5.5 | G46-6 | BD Biosciences | AB_1727529 |
| CD45RA | Anti-human | FITC | HI100 | BD Biosciences | AB_2621694 |
| CCR6 | Anti-human | BUV737 | 11A9 | BD Biosciences | AB_2870109 |
| CD49d | Anti-human | BV711 | 9F10 | Biolegend | AB_2738049 |
| CD29 | Anti-human | APC | TS2/16 | Biolegend | AB_1659685 |
| CD11a/CD18 | Anti-human | BV421 | m24 | Biolegend | AB_2716067 |
| CD4 | Anti-human | PE-CY7 | OKT4 | Biolegend | AB_2573327 |
| CXCR3 | Anti-human | BV605 | G025H7 | Biolegend | AB_2562200 |
| CXCR3 | Anti-human | BV711 | G025H7 | Biolegend | AB_2563532 |
| CD3 | Anti-human | PerCPcy5.5 | UCHT1 | Biolegend | AB_2033956 |
| CD45RA | Anti-human | APC | HI100 | Biolegend | AB_2536108 |
| CCR6 | Anti-human | BV650 | G034E3 | Biolegend | AB_2563869 |
| CCR7 | Anti-human | BV421 | G043H7 | Biolegend | AB_10915137 |
| IFNg | Anti-human | BUV395 | B27 | BD Biosciences | AB_2738277 |
| IL-17A | Anti-human | BV650 | N49-653 | BD Biosciences | AB_2738402 |
| GM-CSF | Anti-human | PE | BVD2-21C11 | BD Biosciences | AB_395440 |
| TNFa | Anti-human | BV785 | Mab11 | Biolegend | AB_2565858 |
| CXCR4 | Anti-human | BV421 | 12G5 | BD Biosciences | AB_11153865 |
