# Supplementary figures and images for: Pro‐Inflammatory c‐Met+ CD4 T Cells in Multiple Sclerosis
Source: Ann Neurol. 2025 Sep 26;99(1):261–73. doi: 10.1002/ana.78035 (PMC12946607; doi:10.1002/ana.78035)

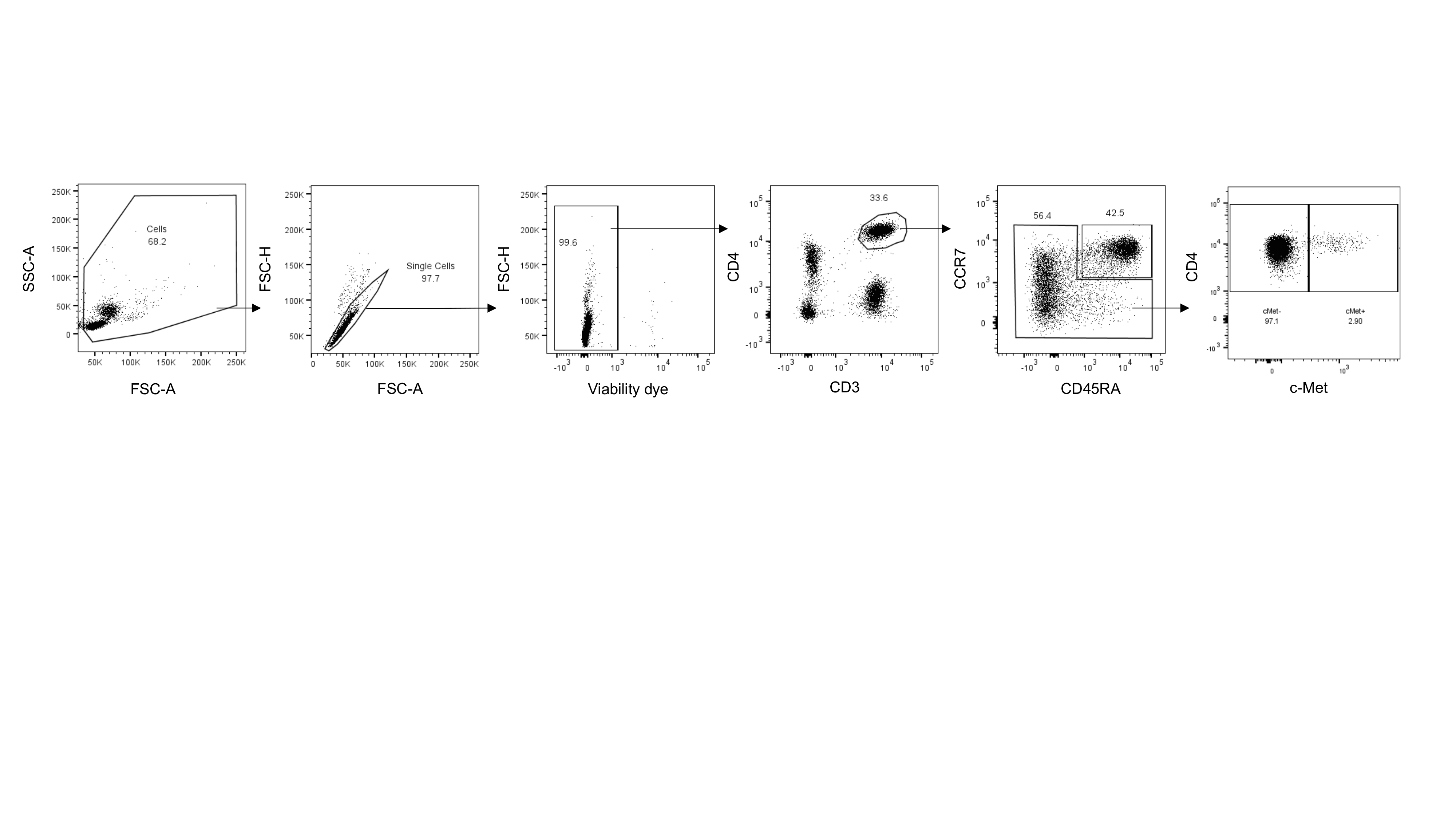

Supplement: Supplementary file 2 — Figure S1. Representative gating strategy of blood c‐Met+ memory CD4 T cells. Freshly isolated PBMC were stained with antibodies directed against CD3, CD4, CCR7, CD45RA and c‐Met to identify c‐Met+ and c‐Met− among memory (non‐CCR7+CD45RA+) CD4 T cells. [file ANA-99-261-s006.tif]

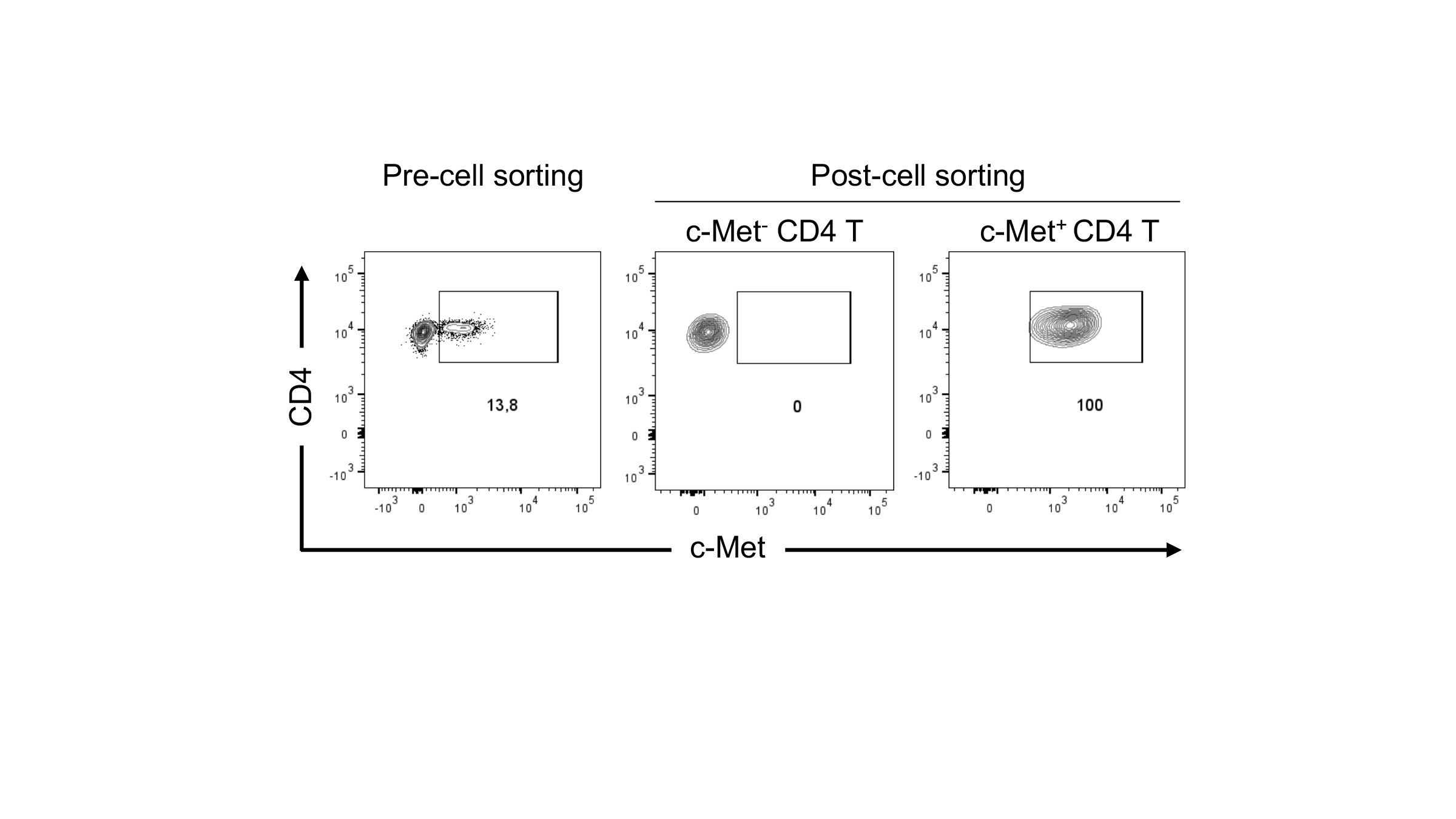

Supplement: Supplementary file 3 — Figure S2. Representative purity of fluorescence‐activated cell sorting (FACS) of c‐Met+ and c‐Met− CD4 T cells. Freshly isolated PBMC were stained with antibodies directed against CD3, CD4 and c‐Met to sort c‐Met+ and c‐Met− CD4 T cells. Sorted populations were examined for their purity, which routinely exceeded >95%. [file ANA-99-261-s003.tif]

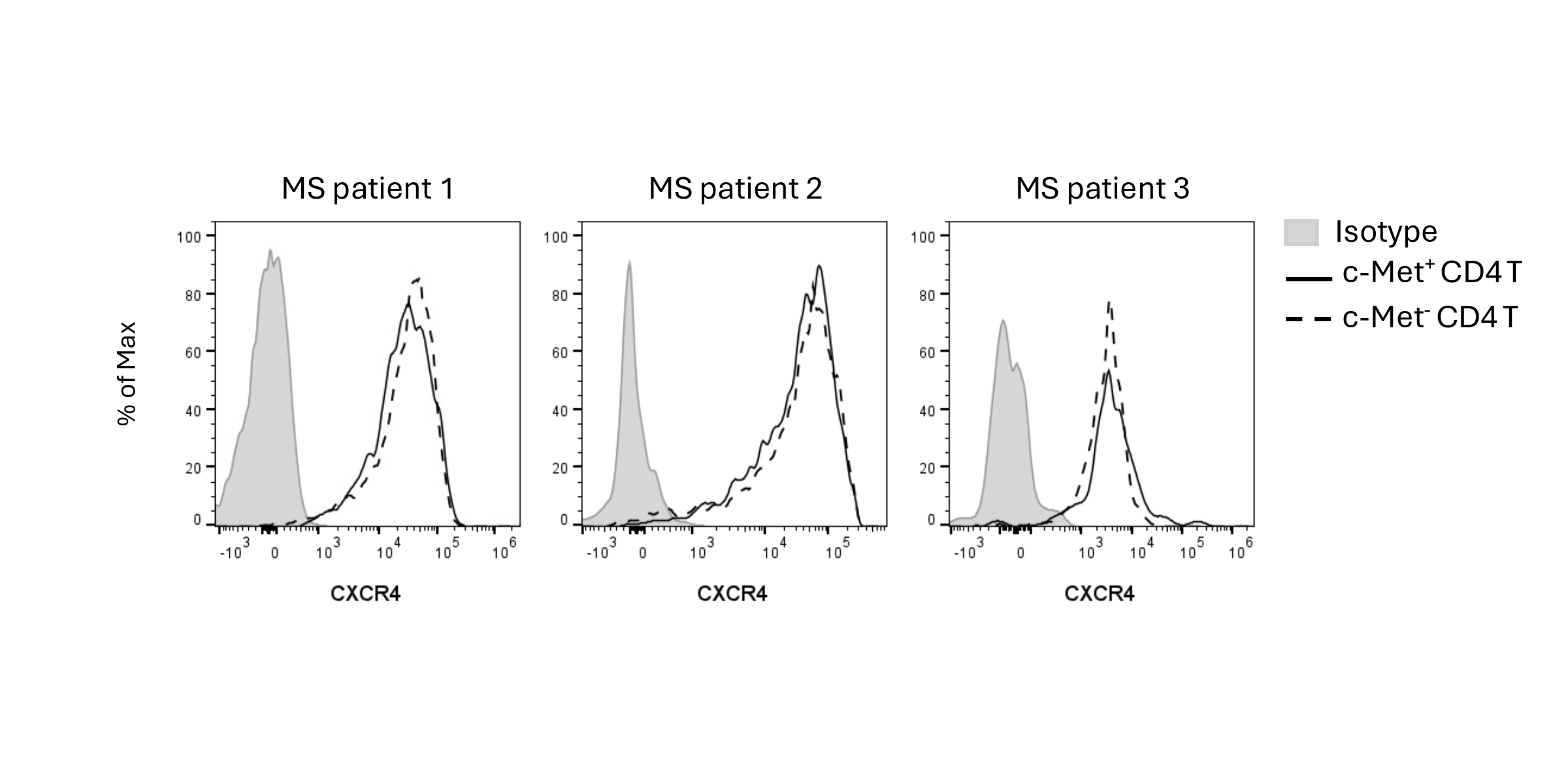

Supplement: Supplementary file 4 — Figure S3. Similar CXCR4 expression on c‐Met+ and c‐Met− CD4 T cells in the blood of MS patients. CXCR4 expression was measured on the surface of c‐Met+ (solid line) and c‐Met− (dotted line) CD4 T cells in cryopreserved PBMC from three untreated MS patients. An isotype antibody (shaded gray) was used as a control for CXCR4 staining. [file ANA-99-261-s002.tif]

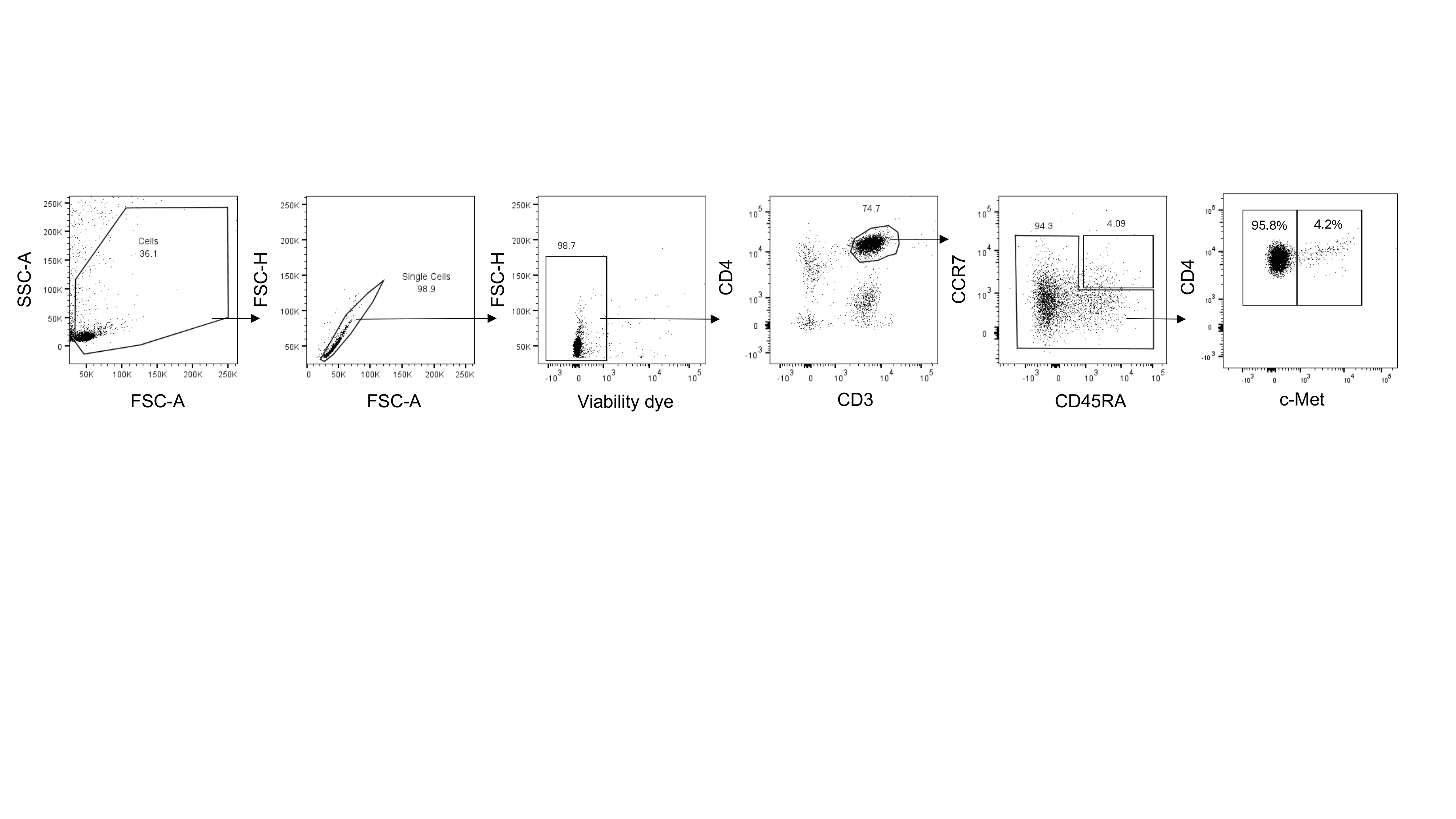

Supplement: Supplementary file 5 — Figure S4. Representative gating strategy of CSF c‐Met+ memory CD4 T cells. Fresh CSF cells were stained with antibodies directed against CD3, CD4, CCR7, CD45RA and c‐Met to identify c‐Met+ and c‐Met− among memory (non‐CCR7+CD45RA+) CD4 T cells. [file ANA-99-261-s001.tif]

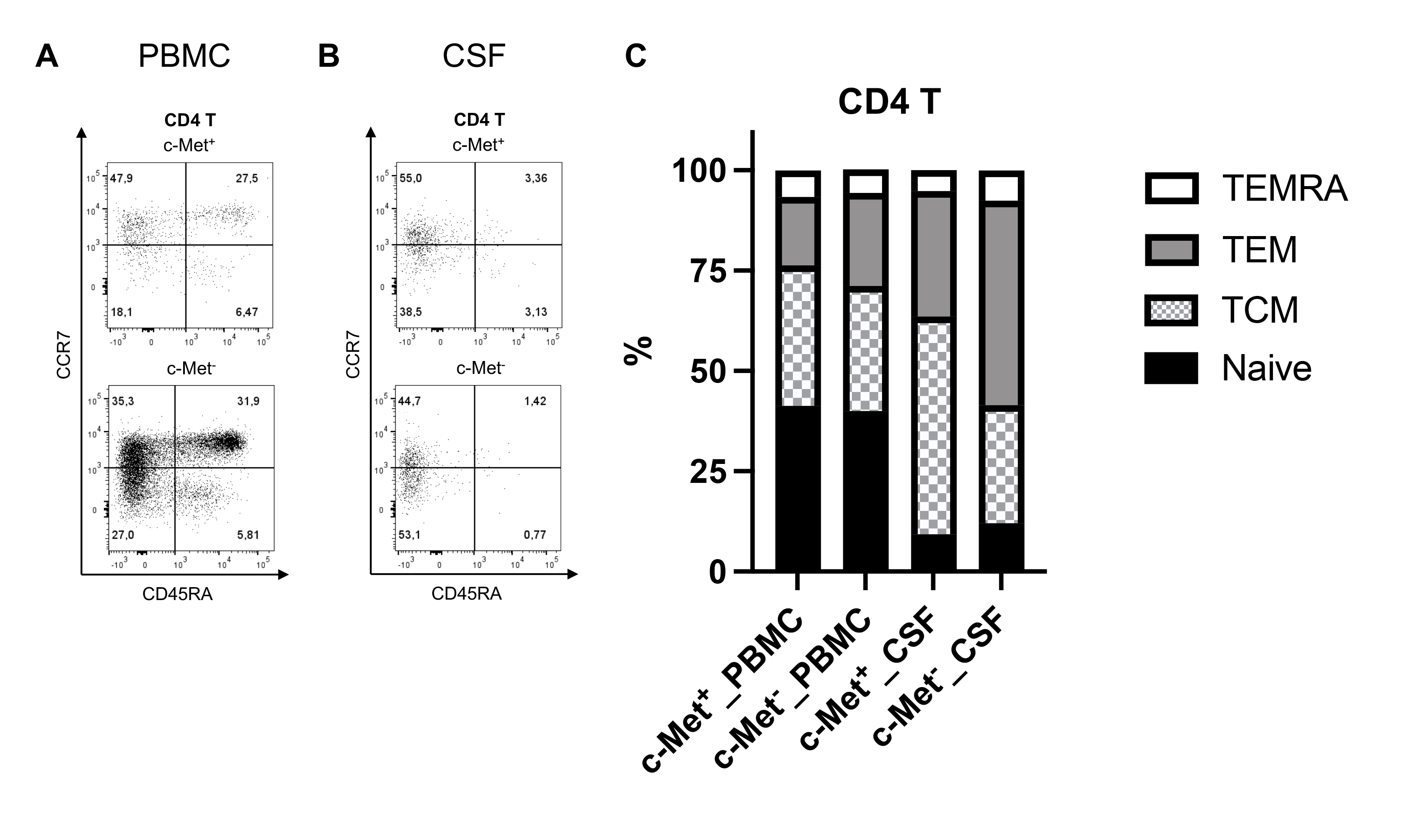

Supplement: Supplementary file 6 — Figure S5. Distribution of naive and memory subsets among c‐Met+ and c‐Met−CD4 T cells in the blood and CSF of MS patients. Representative staining of CCR7 and CD45RA on c‐Met+ and c‐Met− CD4 T cells in PBMC (A) and CSF (B), defining CCR7+CD45RA+ (naive), CCR7+CD45RA− (TCM), CCR7−CD45RA− (TEM) and CCR7−CD45RA− (TEMRA). (C) Relative frequency of naive and memory CD4 T cell subsets among c‐Met+ and c‐Met− CD4 T cells in paired PBMC and CSF samples obtained from MS patients (n = 15). [file ANA-99-261-s004.tif]
